# Decreased thyroid follicle size in dwarf mice may suggest the role of growth hormone signaling in thyroid growth regulation

**DOI:** 10.1186/1756-6614-5-7

**Published:** 2012-08-16

**Authors:** Adam Gesing, Andrzej Bartke, Michal M Masternak, Andrzej Lewiński, Małgorzata Karbownik-Lewińska

**Affiliations:** 1 Department of Oncological Endocrinology, Chair of Endocrinology and Metabolic Diseases, Medical University of Lodz, Lodz, Poland; 2 Department of Internal Medicine, Geriatrics Research, Southern Illinois University School of Medicine, Springfield, IL, USA; 3 College of Medicine, Burnett School of Biomedical Sciences, University of Central Florida, Orlando, FL, USA; 4 Institute of Human Genetics, Polish Academy of Sciences, Poznań, Poland; 5 Department and Chair of Endocrinology and Metabolic Diseases, Medical University of Lodz, Lodz, Poland; 6 Department of Endocrinology and Metabolic Diseases, Polish Mother’s Memorial Hospital – Research Institute, Lodz, Poland

**Keywords:** Ames dwarf mice, GHRKO mice, Thyroid follicle, Inner follicular surface area, Inner follicular perimeter, Follicular epithelium thickness

## Abstract

**Background:**

Altered somatotrophic signaling is among the most important potential mechanisms of extended longevity. Ames dwarf (df/df) mice are homozygous for mutation at the Prop-1 gene, leading to a lack of growth hormone (GH), prolactin and thyroid stimulating hormone (TSH). Mice homozygous for targeted disruption of the growth hormone receptor/growth hormone binding protein gene are known as GH receptor knockout (GHRKO) mice or “Laron dwarf”. Both, df/df and GHRKO mice, are characterized by reduced body size, low plasma insulin and insulin-like growth factor-I (IGF-I), remarkably extended longevity, and severe (in df/df mice) or mild (in GHRKO mice) thyroid hypofunction. Recently, by crossing df/df and GHRKO mice, double-mutant Ames dwarf/GHRKO (df/KO) mice were created. Interestingly, these mice are smaller than Ames dwarfs or GHRKOs, and also have reduced insulin and IGF-I levels. The aim of the study was to investigate if and to what extent certain thyroid morphological parameters, such as inner follicular surface area, inner follicular perimeter, as well as the follicular epithelium thickness are changed in the examined dwarf mice.

**Methods:**

This quantification was performed in thyroids collected from df/df, GHRKO and df/KO female mice, at approximately 5–6 months of age. We used a computerized plotting programme that combines a live microscopic image of the slide with an operator-generated overlay.

**Results:**

Inner follicular surface area and inner follicular perimeter were decreased in all examined kinds of dwarf mice as compared to normal animals. Furthermore, decreases in these two parameters were more pronounced in df/df and df/KO than in GHRKO mice. Concerning the follicular epithelium thickness, only a tendency towards decrease of this parameter was found in all three kinds of dwarf mice.

**Conclusions:**

Parameters characterizing thyroid follicle size are decreased in all three examined models of dwarf mice, which may explain decreased thyroid hormone levels in both basal mutants (Ames dwarfs and GHRKOs). df/df mutation seems to predominate over GHRKO genetic intervention concerning their effects on thyroid growth. Beside TSH, also GH signaling seems to constitute a crucial element in the regulation of thyroid growth and, possibly, function.

## Background

The thyroid gland plays a crucial role, through thyroid hormones (TH) synthesis, in the regulation of various physiological processes, including development of the central nervous system, skeletal growth or heat production. The synthesis of TH is regulated by hypothalamus-pituitary-thyroid axis in the negative feedback loop. Also concerning the regulation of thyroid growth processes, the hypothalamus-pituitary-thyroid axis constitutes the main mechanism, with thyroid stimulating hormone (TSH) being the strongest stimulatory factor for thyroid hyperplasia and proliferation. However, the role of other factors, such as, for example, insulin-like growth factor-I (IGF-I) or epidermal growth factor (EGF), in thyroid development, growth and function, is considered and, partially, documented [1-4]. The possible strong contribution of factors other than “classical” to the regulation of thyroid growth and function is further supported by the following observation. It has been recently found that iodine-deficient diet does not induce thyroid enlargement, whereas decreasing thyroxine secretion in Slc26a4-null mutant mice, being an experimental model for Pendred syndrome [5].

Concerning external factors, iodine deficiency is proven to be the most common cause of thyroid enlargement, leading to goiter formation [1]. Importantly, a high prevalence of goiter is observed in patients with acromegaly [characterized by increased growth hormone (GH) synthesis and elevated IGF-I level] [6]. Furthermore, the increased prevalence of thyroid cancer in acromegalic patients is observed [7,8]. In turn, the transgenic mice over-expressing GH, which may constitute an experimental model of acromegaly, are characterized by increased adult body mass and drastically shortened lifespan [9].

In agreement with the above, experimental evidence suggests that reduced GH signaling may contribute to lifespan extension. Therefore, several types of laboratory animals with disruption of somatotrophic axis are used in aging research. The animals with altered somatotrophic signaling are, among others, Ames dwarf (df/df) mice. These rodents are homozygous for a spontaneous recessive mutation at the transcription factor Prop-1, leading to a lack of GH (with undetectable plasma level of IGF-I), prolactin (Prl) and TSH [10]. The absence of GH, Prl and TSH is a consequence of hypofunctional development of the anterior pituitary and a failure of differentiation of lactotrophs, somatotrophs and thyrotrophs [10]. Interestingly, these dwarf mice live much longer than their normal siblings [11] and are characterized by delayed aging [12], small body size and delayed puberty [13]. Moreover, Ames dwarfs have decreased plasma glucose and insulin levels [13], enhanced insulin sensitivity [14], reduced body core temperature [15], enhanced mitochondrial function [16] and importantly, they are severely hypothyroid [13].

Another experimental model characterized by prolonged lifespan results from genetic intervention which relies on the targeted disruption of the GH receptor/GH binding protein gene (*Ghr/bp* gene) [17]. Mice homozygous for this mutation are known as GH receptor/GH binding protein knockout (GHRKO; *Ghr/bp* −/−) mice or “Laron dwarf” [17]. These GH-resistant mice also live longer than their normal siblings and are characterized by reduced mass and body size, undetectable level of GH receptors, high level of serum GH, greatly reduced plasma levels of insulin and IGF-I, enhanced insulin sensitivity, reduced oxidative damage and improved oxidative stress resistance [13,17-26]. Furthermore, GHRKO mice have lower incidence and delayed onset of fatal neoplastic diseases [27]. The mice with disrupted GH receptor are also characterized by decreased levels of pro-apoptotic factors [28-30] and increased levels of key regulators of mitochondrial biogenesis [31,32]. Importantly, GHRKO mice may have decreased thyroid hormone levels [33], however the results are not consistent [33,34]. Presumably, the differences in levels of thyroid hormones in GHRKO mice may be gender- and age-dependent [33,34]. Similar observations, showing gender-specific changes in thyroid hormones levels in humans during aging, were reported by Suzuki et al. [35].

Interestingly, by crossing df/df and GHRKO mice, double-mutant Ames dwarf/GHRKO (df/KO) mice have been recently created. These mice lacking circulating GH and GH receptor are smaller than Ames dwarfs or GHRKOs, and also have reduced insulin and IGF-I levels [36].

The aim of the study was to investigate if and to what extent certain thyroid morphological parameters, such as inner follicular surface area, inner follicular perimeter, as well as the follicular epithelium thickness are changed in the examined dwarf mice.

## Methods

### Animals

Normal mice (N; n = 5), Ames dwarf mice (df/df; n = 5), growth hormone receptor knockout mice, derived from animals kindly provided by Dr. J.J. Kopchick (Ohio University, Athens, OH, USA) (GHRKO; n = 7) and double-mutant Ames dwarf/GHRKO mice (df/KO; n = 7) (all females) were bred and maintained under temperature- and light-controlled conditions (22 ± 2°C, 12 hr light/12 hr dark cycle). At 5–6 months of age, the animals were anesthetized and euthanized by decapitation. After decapitation, thyroid lobes were collected and fixed in 10% formalin for 6 hours, and, next, in 70^o^ alcohol. Then, the procedure of embedding of thyroid lobes in paraffin wax (using 95^o^ alcohol, aniline, carboxylene, xylene and, finally, paraffin) was performed. Next, the paraffin sections (4 μm thick), after deparaffinating, were stained with hematoxylin and eosin.

### Assessment of thyroid morphological parameters

In order to evaluate the inner follicular surface area, inner follicular perimeter and follicular epithelium thickness, a computerized microscopy analysis system (Neurolucida, MicroBright Field Inc, Colchester, VT, USA) was used. This programme combines a live microscopic image of the slide with an operator-generated overlay.

### Statistical analysis

The data were statistically analysed, using Student’s unpaired *t* test or the one-way analysis of variance (ANOVA), followed by Student-Newman-Keuls’ test. Statistical significance was determined at the level of *p<*0.05. The results, obtained from the right and left lobes, are jointly depicted in the figures and expressed as means ± SEM. All statistical calculations were conducted using SPSS version 17.0 (SPSS, Chicago, IL) with α=0.05. All graphs were made using Prism 4.02 (GraphPad Software, San Diego, CA).

## Results

**Figure 1:**
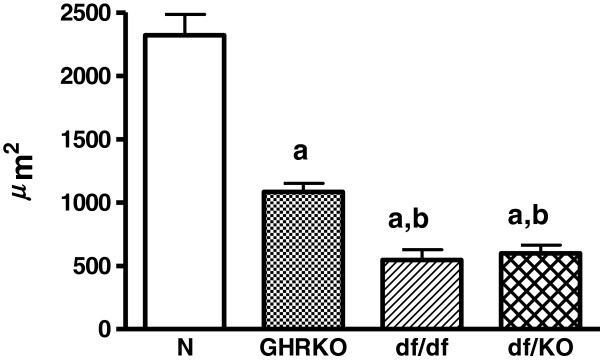
**Inner follicular surface area.** Inner follicular surface area in normal (N), growth hormone receptor/binding protein knockout (GHRKO), Ames dwarf (df/df) and double-mutant Ames dwarf/GHRKO (df/KO) mice. Values are means ± SEM. a – p<0.001 vs. normal mice; b – p<0.05 vs. GHRKO mice.

**Figure 2:**
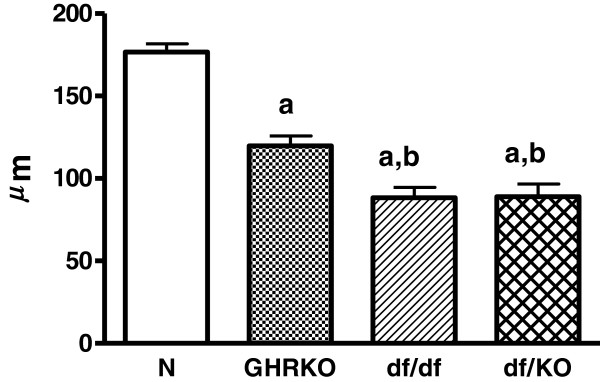
**Inner follicular perimeter.** Inner follicular perimeter in normal (N), growth hormone receptor/binding protein knockout (GHRKO), Ames dwarf (df/df) and double-mutant Ames dwarf/GHRKO (df/KO) mice. Values are means ± SEM. a – p<0.001 vs. normal mice; b – p<0.04 vs. GHRKO mice.

**Figure 3:**
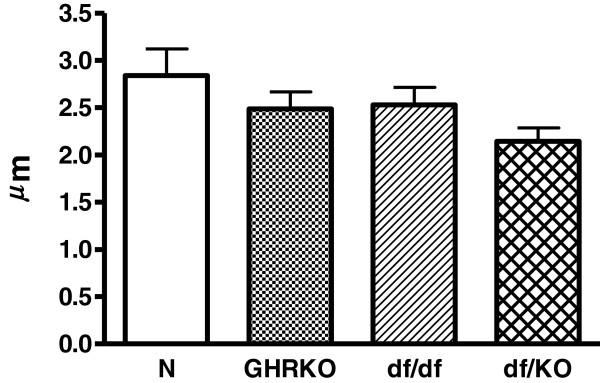
**Follicular epithelium thickness.** Follicular epithelium thickness in normal (N), growth hormone receptor/binding protein knockout (GHRKO), Ames dwarf (df/df) and double-mutant Ames dwarf/GHRKO (df/KO) mice. Values are means ± SEM.

Inner follicular surface area was decreased in Ames dwarf (df/df), GHRKO and double-mutant Ames dwarf/GHRKO mice (df/KO) as compared to normal animals (p<0.001 all) (Figure 1). Moreover, this morphological parameter was also decreased in df/df and df/KO mice in comparison with GHRKO dwarfs (p = 0.034, p = 0.04, respectively) (Figure 1). No changes in inner follicular surface area between df/df and df/KO mice were observed (Figure 1). Similarly to inner follicular surface area, also inner follicular perimeter was decreased in df/df, GHRKO and df/KO mice as compared to normal animals (p<0.001 all) (Figure 2). The inner follicular perimeter was also decreased in df/df and df/KO mice in comparison with GHRKO animals (p = 0.039, p = 0.028, respectively) (Figure 2). Furthermore, this parameter did not differ between df/df and df/KO mice (Figure 2). Concerning the follicular epithelium thickness, only a tendency towards decrease of this parameter in all kinds of the examined dwarf mice was found (Figure 3).

## Discussion

We have expectedly observed in our study that the inner follicular surface area and the inner follicular perimeter were decreased in all examined kinds of dwarf mice. At least two causes of such results can be considered. First, the decrease of these parameters may be a direct consequence of smaller sizes and masses of Ames dwarf (df/df), GHRKO and df/KO mice as compared to normal animals. In accordance with our observation, Bartke [37] reported previously that the thyroid glands of Ames dwarfs and Snell mice (another kind of dwarf mice, characterized by a mutation at the transcription factor Pit-1 [38]) were greatly reduced in size, the follicles were small, and in the center of each lobe a considerable amount of the tissue was not organized into follicles. Similarly, Cordier et al. [39] demonstrated reduced follicular parameters (e.g. follicular surface and volume) in Snell mice as compared to normal mice. According to such an explanation, even lower values of measured parameters should be observed in double mutants, i.e. df/KO, as they are characterized by smaller body mass and size when compared to df/df or GHRKO mice [36]. However, both, the inner follicular surface area and the inner follicular perimeter in double mutants df/KO were the same as in case of df/df mice. This means that the additional experimental intervention, namely GHRKO, did not add anything concerning these parameters. Therefore, the lack of differences in the examined thyroid morphological parameters between df/KO and df/df mice suggests that some additional factors could exist, being responsible for such result, and presumably it may be linked to thyroid secreting function. Thus, the second cause may relate to certain factors which decrease thyroid function much stronger in df/df mice [13] than in GHRKO animals [33]. Thus, on the basis of our results we can suggest that df/df mutation predominates over GHRKO genetic intervention concerning their effects on thyroid growth and, possibly, function. However, further analysis of thyroid function in df/KO mice is needed.

It is clear that the interruption of somatotrophic axis (at different levels) is responsible for the smaller body mass, smaller body size and smaller size of different organs, thyroid included, in dwarf mice. In this context, it is worth stressing that IGF-I acts also locally in the thyroid gland, e.g. via enzymes involved in DNA synthesis [40]. Furthermore, IGF-I may contribute to thyroid cancers development [41]. Therefore, the somatotrophic signaling can also be reduced in this endocrine gland in dwarf mice, what may, presumably, lead to the decrease of the examined thyroid morphological parameters.

Furthermore, decreased inner follicular surface area and inner follicular perimeter in dwarf mice may suggest smaller amount of colloid in the thyroid gland. It is known that decreased colloid content is observed in hyperthyroidism. In contrast, df/df and GHRKO mice are characterized, as was reported above, by severe (in df/df mice) or mild (in GHRKO mice) thyroid hypofunction. However, one should conclude that results from the present study can not be directly extrapolated into the studies in humans. Even contradictory results have been obtained between experimental studies in humans. For example, whereas decreased oxidative stress was found in dwarf mice [13], huge oxidative damage to membrane lipids was reported in GH-deficient patients [42,43], the phenomenon which was partially reversed by GH replacement [43].

Interestingly, both GH and IGF-I increased the iron-induced lipid peroxidation (LPO) in porcine thyroid homogenates [44]. On the other hand, GH and IGF-I may prevent the iron-induced oxidative damage in other tissues [45]. These results suggest that somatotrophic axis may reveal either pro-oxidative or anti-oxidative action, depending on the current redox status. This, in turn, may contribute to the regulation of thyroid growth and function [46].

The results of the present study are important not only in the context of longevity. There is a strong relationship between thyroid function, GH–IGF-I axis and bone linear growth in animals and humans. It is well known that thyroid hormones are indispensable for bone linear growth. Additionally, untreated hypothyroidism unfavourably affects the results of replacement therapy in GH-deficient patients and also it disturbes the results during diagnosis [47,48]. Thus, further studies, morphological evaluation included, on the thyroid function in subjects with disturbed GH–IGF-I axis would be of great importance.

Taking into account all above observations, one should hypothesize that GH signaling may contribute to thyroid growth and/or function, and that thyroid function (and/or morphological development) may be somehow affected by somatotrophic axis, although various other factors contributing to the development of thyroid gland are also known.

## Conclusions

Parameters characterizing thyroid follicle size are decreased in all examined kinds of dwarf mice, which may explain decreased thyroid hormone levels in both basal mutants (Ames df/df dwarfs and GHRKOs). df/df mutation seems to predominate over GHRKO genetic intervention concerning their effects on thyroid growth. Beside TSH, also GH signaling seems to constitute a crucial element in thyroid growth and, possibly, function.

## Competing interests

The authors declare that they have no competing interests.

## Authors’ contributions

AG designed and performed the experiment, assessment of thyroid morphological parameters, data analyses and interpretation and wrote the manuscript. MKL supervised preparation of the manuscript and was involved in the interpretation of the results. MMM and AB contributed to the experimental design. MMM, AL and AB were involved in the interpretation of the results and review of the manuscript. All authors read and approved the final manuscript.
